# Genistein Modulates Signaling Pathways and Targets Several Epigenetic Markers in HeLa Cells

**DOI:** 10.3390/genes10120955

**Published:** 2019-11-21

**Authors:** Madhumitha Kedhari Sundaram, Sreepoorna Unni, Pallavi Somvanshi, Tulika Bhardwaj, Raju K. Mandal, Arif Hussain, Shafiul Haque

**Affiliations:** 1 School of Life Sciences, Manipal Academy of Higher Education, P.O. Box 345050 Dubai, UAE; madhumithakedhari@gmail.com; 2 Department of Life and Environmental Sciences, College of Natural and Health Sciences, Zayed University, P.O. Box 19282 Dubai, UAE; Sreepoorna.Unni@zu.ac.ae; 3 Department of Biotechnology, TERI School of Advanced Studies, 10, Institutional Area, Vasant Kunj, New Delhi-110070, India; psomvanshi@gmail.com (P.S.); tulika15june@gmail.com (T.B.); 4 Research and Scientific Studies Unit, College of Nursing and Allied Health Sciences, Jazan University, Jazan-45142, Saudi Arabia; rajmandalbiot@gmail.com

**Keywords:** genistein, epigenetic, tumour suppressor gene, cancer

## Abstract

Background: Several epigenetic changes are responsible for transcriptional alterations of signaling pathways and tumour suppressor genes (TSGs) contributing to carcinogenesis. This study was aimed to examine the effect of the phytochemical, genistein on various molecular targets in HeLa cells. Methods: Quantitative PCR was used to analyze the expression of various molecular targets. Biochemical assays were employed to study the epigenetic enzymes. To correlate the transcriptional status of the selected TSGs and epigenetic modulation, their promoter 5’CpG methylation levels were evaluated by quantitative methylation array followed by methylation specific restriction digestion. Results: The expression of several genes involved in the cell cycle regulation, migration, inflammation, phosphatidylinositol 3-kinase (PI3K) and mitogen activated kinase-like protein (MAPK) pathway were found to be modulated including *CCNB1, TWIST1, MMP14, TERT, AKT1, PTPRR, FOS* and *IL1A*. Genistein modulated the expression of DNA methyltransferases (DNMTs), histone deacetylases (HDACs), histone methyltransferases (HMTs), demethylases, and histone phosphorylases. Furthermore, genistein decreased the activity of DNMTs, HDACs, and HMTs and reduced global DNA methylation levels. Promoter methylation of several TSGs, including *FHIT, RUNX3, CDH1, PTEN*, and *SOC51*, was lowered with corresponding transcriptional increase. Network analysis indicated similar effect of genistein. Conclusion: This study presents a comprehensive mechanism of action of genistein showcasing effective epigenetic modulation and widespread transcriptional changes resulting in restoration of tumour suppressor gene expression. This study corroborates the development of genistein as a candidate for anti-cancer therapy.

## 1. Introduction

Carcinogenesis involves both genetic and epigenetic changes, which can alter the expression of genes that are central to the development of cancer. Epigenetic modifications are alterations occurring in the genetic material that do not cause any change in the nucleotide sequence; but may cause conformational modifications in the DNA [1]. While, epigenetic mechanisms are required for normal physiological development and gene expression, aberrant alterations are linked to the development of carcinogenesis [2,3]. The epigenome is characterized by DNA methylation and posttranslational modifications of histone proteins leading to alterations in chromatin structure and gene expression. DNA methyltransferases (DNMTs) namely, DNMT1, DNMT3A, and DNMT3B, which are responsible for the methylation of CpG islands in DNA and suppression of gene expression are counteracted by demethylases [4,5]. Histone deacetylases (HDACs) mediate deacetylation of histones leading to silencing of gene expression and are countered by the action of histone acetylases (HATs) [6]. DNA methylation and histone acetylation are interlinked [7]. The modulation of gene expression also occurs via the action of various histone modifiers including histone phosphorylases, histone methyltransferases, histone demethylases and histone ubiquitinases. DNA methyltransferases (DNMTs), HDACs and other histone modifiers are found to be overexpressed in various cancers, thus changing the subtle equilibrium and allowing for tumor development and progression. The changes in the levels of promoter methylation and histone acetylation of tumor suppressor genes (TSGs) are an outcome of the disturbance in enzyme equilibrium and ultimately results in cancer development [8]. Together, they alter chromatin accessibility and transcription factor binding site availability, which then accounts for silencing of tumor suppression genes involved in the regulation of cellular processes like cell propagation, cell-cycle maintenance and apoptosis [9,10]. Epigenetic changes are reversible, hence drugs that target these enzymes play a critical role in restoring epimutations in malignant cells and are potential candidates for cancer therapy [11]. Epigenetic inhibitors are being established and checked for their potency in cancer therapy, however, their widespread use is curtailed due to lack of specificity, brief action time and negative impact on non-tumour cells [12]. Therefore, there is an urgent need to identify safe and efficient epigenetic modulators. Epidemiological studies have shown that diets rich in plant polyphenols defend against cancer initiation and progression [13]. Dietary polyphenols exhibit the capacity for targeting inflammation, proliferation, induce apoptosis and work as potential adjuvants to chemotherapeutic drugs [14,15]. Their chemopreventive potential may also be attributed to their restoration of TSG expression via epigenetic modulation [16]. In this study, we explore the effect of genistein on various signaling pathways, molecular targets, epigenetic modulators and their functional impact on TSG expression. Further, *in-silico* network analysis was performed in order to validate the essentiality and biological significance of genistein on several underlying pathways.

## 2. Materials and Methods

### 2.1. Cell Line and Cell Culture

Human cervical carcinoma cells (HeLa) cells were maintained in Dulbecco’s Modified Eagle’s Medium (Sigma, St Louis, MO, USA). Media was supplemented with fetal bovine serum (10%) (Sigma, St Louis, MO, USA) as well as Pen-strep (100×) (Sigma, St Louis, MO, USA). Cells were placed in an incubator at 37 °C, suffused with 5% CO_2_ and sufficient humidity.

### 2.2. Preparation of Genistein

Genistein (Sigma, St Louis, MO, USA) was prepared into a 10 mM stock using DMSO and stored at −20 °C. 1 mM genistein was made in a complete medium and used as the working concentration. A range of concentrations were tested in advance by MTT (3-(4, 5-dimethylthiazol-2-yl)-2, 5-diphenyltetrazolium bromide) assay and 100 μM for 24 h was identified as the EC_50_ value. For this study, sub-lethal dose of 50 µM genistein was used for all the assays.

### 2.3. Expression Analysis of Various Genes Involved in Tumorigenesis and Cancer Related Pathways

A total of 2x10^6^ cells were plated and treated with 50 µM genistein for 48 h. Gen Elute Mammalian Genomic Total RNA Kit (Sigma, St Louis, MO, USA) was used to obtain total RNA from genistein treated and untreated HeLa cells. RT-PCR Kit (ABI, Waltham, MO, USA) was used to synthesize cDNA which was subsequently used for the array. TaqMan-based array was customized with primers specific for several genes involved in signal transduction pathways as well as TSGs. PCR array was run on QuantStudio3 and analyzed by the Comparative Delta Delta Ct method (ΔΔCT method) using DataAssist^TM^ software v3.01 (ThermoFisher, Waltham, MO, USA) with global normalization. RQ signifies the relative fold change in gene expression of treated sample with respect to untreated control. The statistical significance was calculated as per the mean of three experiments using two-tailed *t*-test with *p* ≤ 0.05.

### 2.4. DNA Methyltransferase Activity Assay

Untreated HeLa cells were processed for obtaining the nuclear extract by using EpiQuikTM nuclear extraction kit (Epigentek, New York, USA) following the manufacturer’s protocol. Epiquik DNMT activity assay kit (Epigentek, New York, USA) was used to examine the effect of genistein (50 µM) on the activity of DNMT enzymes. Nuclear extract was added to the assay plate along with the buffers and genistein and incubated for 1.5 h at 37 °C. ELISA based detection was performed to quantitate the product formed. The percentage of DNMT inhibition following genistein treatment in contrast to the untreated control was assessed by the following formula, where OD is optical density:

DNMT Inhibition (%) = (1−(Treated Sample OD−Blank)/(Control Sample OD−Blank)) × 100%

### 2.5. Histone Deacetylase Activity Assay

Nuclear extract was obtained from the untreated HeLa cells as mentioned in the previous section. Epiquik HDAC activity assay kit (Epigentek, New York, USA) was used to ascertain the effect of genistein (50 µM) on the activity of HDAC enzymes. Nuclear extract was added to the assay plate along with the buffers and genistein and placed at 37 °C for 1 h. Afterwards, ELISA based detection was performed to quantitate the product formed. The percentage of HDAC inhibition following genistein treatment in contrast to the untreated control was assessed by the following formula, where OD is optical density:

HDAC Inhibition (%) = (1−(Treated Sample OD−Blank)/(Control Sample OD−Blank)) × 100%

### 2.6. Histone Methyltransferase-H3K9 Activity Assay

Nuclear extract was obtained from the untreated HeLa cells as mentioned earlier. The Epiquik histone methyltransferase H3K9 (HMT-H3K9) activity assay kit (Epigentek, New York, USA) was used to observe the effect of genistein (50 µM) on the activity of HMT enzymes. Nuclear extract was added to the assay plate along with the buffers and genistein and incubated for 1.5 h at 37 °C. Further, ELISA based detection was performed to quantitate the product formed. The percentage of inhibition compared with the untreated control was then assessed using the below mentioned formula and plotted as a graph.

HMT H3K9 Inhibition (%) = (1−(Treated Sample OD−Blank)/(Control Sample OD−Blank)) × 100%

### 2.7. Expression Analysis of the Genes Involved in Chromatin Modification

cDNA was prepared as described in the preceding section and used as the template. Human Epigenetic Chromatin Modification Enzymes RT² Profiler PCR Array (Qiagen, Venlo, Netherlands) was used to profile the expression of epigenetic genes involved in methylation of DNA and modification of histones. This includes DNA methyltransferases, demethylases, histone acetylases, deacetylases, methylases, histone phosphorylases and ubiquitinases. Fold(s) change over the untreated control was calculated after normalization with the endogenous gene, glyceraldehyde-3-phosphate dehydrogenase (GAPDH). Statistical significance was estimated using the mean of three experiments and two-tailed *t*-test with *p* ≤ 0.05.

### 2.8. Global DNA Methylation Assay

For this assay, around 2×10^6^ HeLa cells were treated with 50 μM genistein (for 24 and 48 h) and DNA was isolated using GenElute Mammalian Genomic DNA Miniprep Kit (Sigma, St Louis, MO, USA). Following the treatment with 50µM genistein for 24 and 48 h, methylated DNA was bound by 5-mC antibody, which was detected colorimetrically as per the manufacturer’s protocol using MethylFlash™ Methylation DNA Quantification kit (Epigenetek, New York, USA). The optical density values were proportional to the amount of globally methylated DNA. The level of methylation was calculated as per the formula and are expressed as percentage of control and represented in a graph.

Methylation compared to the control DNA (%) = (Mean OD of the treated sample−Blank/Mean OD of the control−Blank) × 100

### 2.9. Detection of Promoter Methylation Using Methylation Array

HeLa cells were treated with 50 µM genistein for 48 h and DNA was extracted using the GenElute Mammalian Genomic DNA Miniprep Kit (Sigma, St Louis, MO, USA) and the EpiTect II DNA Methylation Enzyme Kit (Qiagen Venlo, Netherlands) was used to perform restriction digestion. The assay uses two different restriction endonucleases whose activity depends on whether methylated cytosines are present or absent in the recognition sequence. Restriction digestion using (i) an equal quantity of DNA with no enzyme, (ii) one of the two enzymes, and (iii) a double digest with both enzymes was set up. The products of the four restriction digests where then individually used as the template for qPCR (Human Tumor Suppressor Genes EpiTect Methyl II Signature PCR Array (Qiagen, Venlo, Netherlands). The cycle threshold (Ct) values of each gene, from each digest was used to calculate the extent of methylation.

### 2.10. Statistical Analysis of Experimental Data

All the statistical data have been expressed as means ± standard deviation (SD) of at least 3 experiments. One-way ANOVA followed by two tailed *t*-test was performed (* represents *p* ≤0.05) during the analysis.

### 2.11. In silico Network Analysis of Target Genes

Pool of genes involved in chromatin modification, signal transduction pathways and as tumor suppression were subjected to in-silico network analysis to identify the effect of genistein in modulating underlying disease mechanism by using NetworkAnalyst (https://www.networkanalyst.ca). This platform utilizes network-based approaches to perform gene set enrichment analysis to identify the biological significance among expressed gene sets. Gene set enrichment analysis (GSEA) discerns the biological indications of statistically significant enriched quantitative genomic data. Average fold change (avFC) values of these genes were taken as input parameters to perform network analysis. This methodology overlays gene expression data on experimentally validated gene-to-gene interactions to enrich upregulated and downregulated genes. Further, network topological parameters *viz.* betweenness, degree centrality and expression were computed to identify candidate genes involved in modulation of disease mechanism. 

Network analysis = Network Enrichment Analysis + Network Topological Analysis

### 2.12. Gene Function and Pathway Enrichment Analysis

WebGestalt (http://www.webgestalt.org/), an interactive gene set analysis toolkit was utilized to perform functional enrichment of expressed gene sets. Over-representation analysis (ORA) in combination with network topology-based algorithm perform enrichment analysis of expressed genes of interest. Gene function analysis was used to categorize candidate genes into functional annotations viz. molecular functions, biological pathways and cellular machinery.

## 3. Results

### 3.1. Genistein Modulates the Expression of Genes Involved in Cell Cycle Regulation, Migration, Signaling Pathways and Inflammation

**Figure 1:**
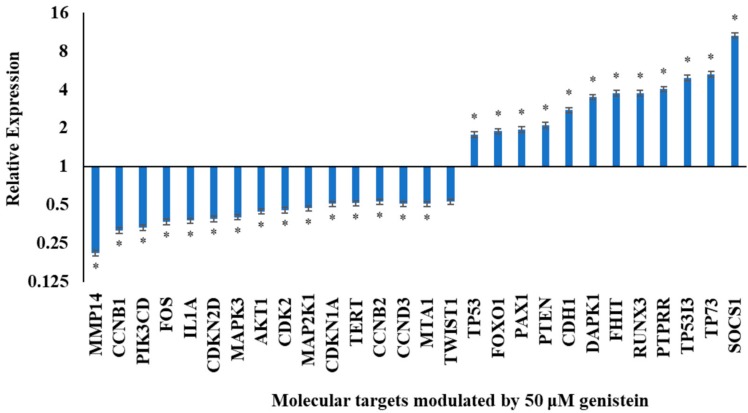
Expression analysis of molecular targets including cell cycle regulators, tumour suppressors and genes involved in phosphatidylinositol 3-kinase (PI3K) and mitogen activated kinase-like protein (MAPK) signaling (*p ≤ 0.05) after 48 h treatment with 50 µM genistein.

In order to understand the effect of genistein on the expression of genes involved in cell regulatory activities and signal transduction, qRT-PCR was performed. Genistein (50 µM) was found to modulate the transcription of several genes as detailed below. The fold change of these variations over untreated control cells was calculated and represented as a graph in Figure 1 and Table 1.

Genistein (50 µM) downregulated *CCNB1, CCNB2* and *CCND3* that are required for the cell to cross G2-M checkpoint. Further, *CDKN1A, CDKN2D, CDK2* and *TERT* were also found to be downregulated. Genistein upregulated *CDH1* and *SOCS1*, while downregulated the pro-migratory genes such as *TWIST1, MTA1* and *MMP14*. The expression of genes in the phosphatidylinositol 3-kinase (PI3K) pathway namely, *PI3KCD* and *AKT* was lowered by genistein. Genistein also modulated mitogen activated kinase-like protein (MAPK) pathway; it reduced the expression of *MAPK3* and *MAP2K1* and increased the expression of *PTPRR. IL1A* and *FOS* are pro-inflammatory molecules whose expression was downregulated by genistein.

### 3.2. Genistein Modulates the Expression of Various Chromatin Modifiers Involved in the Epigenetic Pathway

**Figure 2:**
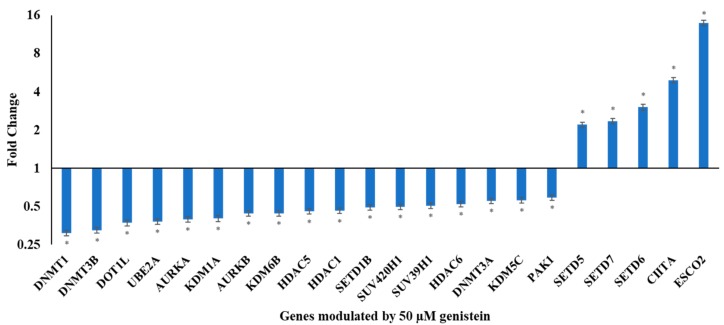
Effect of genistein on genes involved in chromatin modification. RQ plot of genes involved in chromatin modification whose expression in HeLa cells is modulated following treatment with 50 µM genistein for 48 h. Fold change was calculated by ΔΔCT analysis compared to the untreated control after global normalization. The values are means ± SD of three independent experiments. (*p ≤ 0.05).

In order to understand if the observed transcriptional changes could be accounted for the changes in the epigenetic pathway, qRT-PCR of various epigenetic modulators was performed. Genistein downregulated *DNMT1, DNMT3B, DOT1L, UBE2A, AURKA, KDM1A, AURKB, KDM6B, HDAC5, HDAC1, SETD1B, SUV420H1, SUV39H1, HDAC6, DNMT3A, KDM5C* and *PAK1*. Genistein also upregulated several chromatin modifiers including *SETD5, SETD7, SETD6, CIITA* and *ESCO2*. These genes could contribute to the anti-proliferative, anti-metastatic and anti-cancer activity of genistein. The fold changes over untreated control are presented as a graph in Figure 2.

### 3.3. Genistein Inhibits DNMT, HDAC and HMT H3K9 Activity

**Figure 3:**
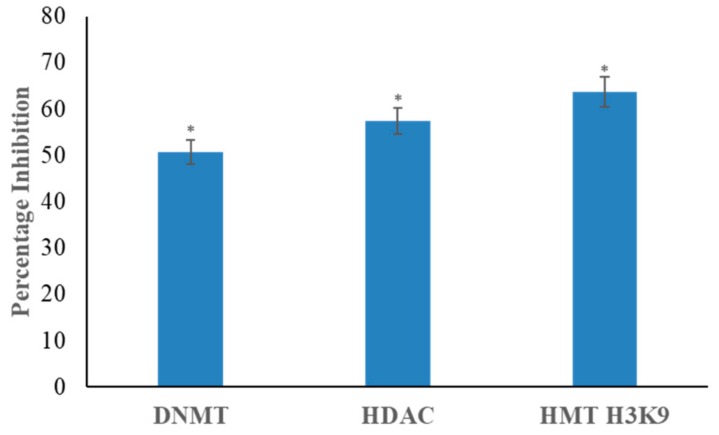
Effect of genistein on the activity of DNA methyltransferase (DNMT), histone deacetylase (HDAC) and histone methyltransferase H3K9 (HMT H3K9) in HeLa cells. 50 µM genistein treated HeLa cells demonstrate significant inhibition of DNMT, HDAC and HMT H3K9 activity. The values are represented in comparison with the untreated control and are means ± SD of three independent experiments (*p ≤ 0.05).

Nuclear extracts incubated with 50 µM genistein brought a significant decline in DNMT activity by 48% in comparison with the untreated control (Figure 3). Genistein (50 µM concentration) was found to inhibit the activity of HDAC enzymes by 57% and HMT H3K9 activity by 63% in the nuclear extract of HeLa cells in comparison with the untreated control (Figure 3). 

### 3.4. Genistein Decreases Global DNA Methylation

**Figure 4:**
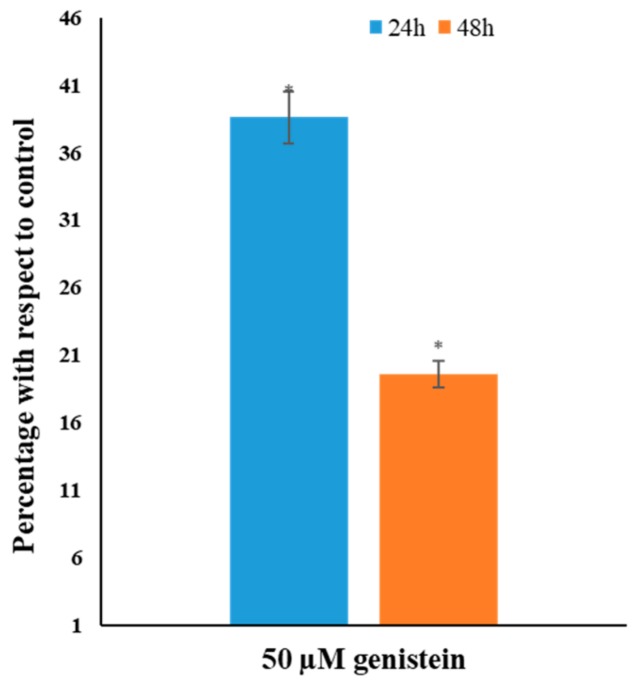
Effect of genistein on global DNA methylation in HeLa cells. Genistein (50 µM) significantly decrease the levels of global DNA methylation in HeLa cells in a time dependent manner. The decrease is methylation level is represented as a percentage of the untreated control. Values are means ± SD of three independent experiments (*p ≤ 0.05).

Genistein induced a steep time dependent decrease in global DNA methylation in HeLa cells. 50 µM genistein treatment for 24 and 48 h reduced DNA methylation to 36 and 19%, respectively, of the untreated control (Figure 4).

### 3.5. Genistein Reduces the Promoter 5’CpG Methylation of Tested Tumour Suppressor Genes

**Figure 5:**
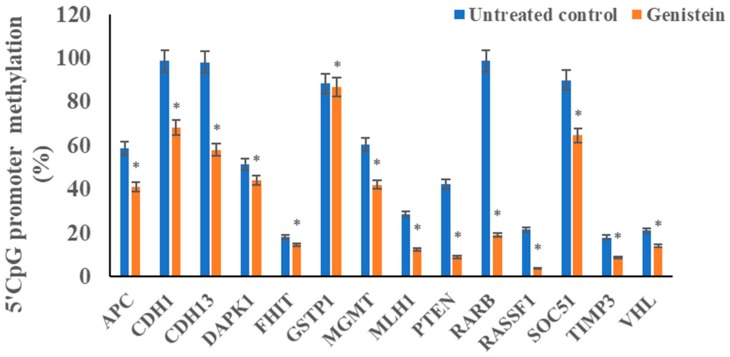
Effect of genistein on 5´ CpG island promoter methylation of tumour suppressor genes (TSGs) in HeLa cells using Human Tumor Suppressor Genes EpiTect Methyl II Signature PCR Array. Genistein (50 µM) significantly decreased the promoter methylation levels in HeLa cells in comparison with the untreated control. The values are mean ± SD of three independent experiments (*p ≤ 0.05).

In order to correlate the modulation of epigenetic enzymes as well as to observed the increase in expression of various tumour suppressor genes, promoter 5’CpG methylation of some genes were studied. Genistein significantly reduced the methylation level of several tumour suppressor genes including *APC, DAPK1, FHIT, PTEN, GSTP1, RARB, RASSF1, CDH1, MLH1, SOC51, TIMP3, CDH13, MGMT* and *VHL* compared to the untreated control (Figure 5).

### 3.6. Genistein Restores the Transcription of Tested Tumour Suppressor Genes

**Figure 6:**
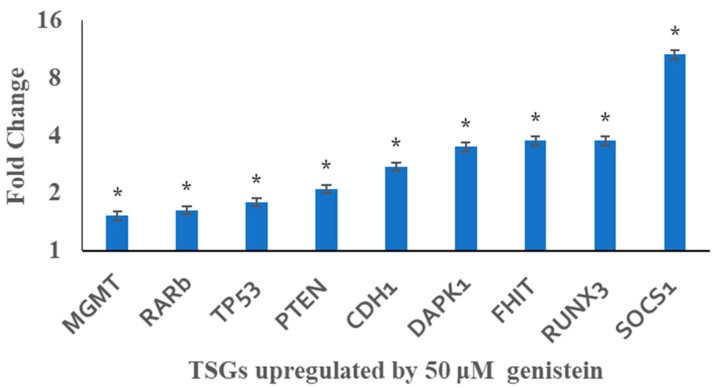
qRT PCR analysis against the effect of genistein on TSG expression in HeLa cells. Genistein (50 µM) significantly increased the levels of TSG expression. Fold change was calculated by ΔΔCT analysis in comparison with the untreated control after normalization with housekeeping gene. The values are means ± SD of three independent experiments (*p ≤ 0.05).

The expression of *TP53, PTEN, CDH1, DAPK1, FHIT, RUNX3*, and *SOCS1* was upregulated by genistein treatment as evidenced by qRT-PCR (Figure 6). This transcription restoration may possibly be correlated with the decrease in their promoter methylation. However, no significant changes were observed in *BRCA1, GSTP1, MLH1, RASSF1, TIMP3*, and *VHL* gene expression at transcript level.

### 3.7. Network Analysis of Expressed Gene Sets

**Figure 7:**
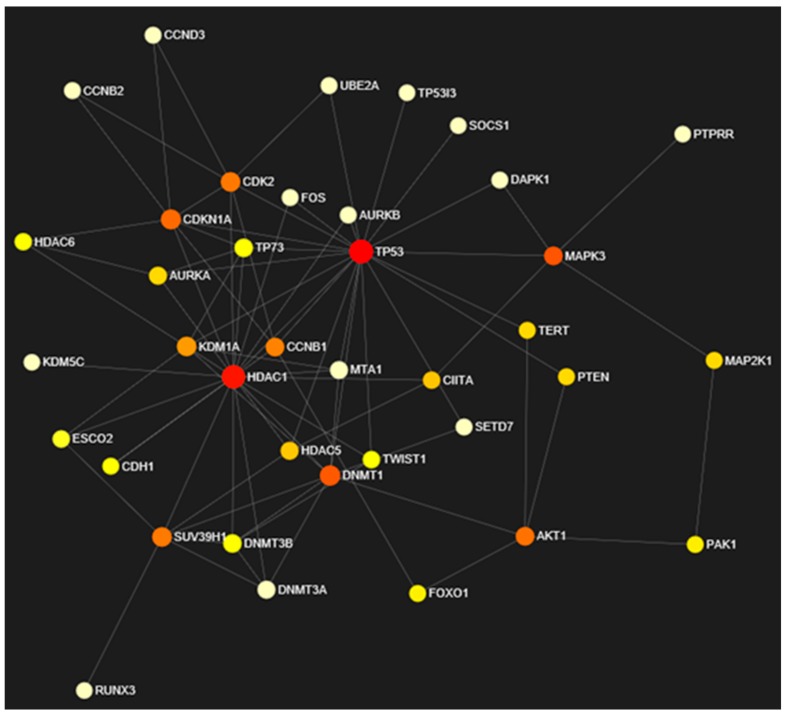
Network based on expression and topological characteristics of input target genes.

Network analysis of input gene sets was performed by considering the expression values and topological parameters of each node within the network. Robustness of the network was estimated by computing (i) *degree centrality*, the number of edged incidents in a graph; (ii) *betweenness*, focused on global network computes number of shortest paths passing through node and (iii) expression, the pattern of nodes in network representing connectivity (Table 2). Graphical format of the network constructed based on the expression is visualized in Figure 7. Among 50 nodes, 38 nodes were found to be essential for network maintenance and robustness. Therefore, topological parameters were computed for essential nodes only in this manuscript. Expression levels in combination with topological characteristics of each node is represented by varying color (*red to white = high expression + essential to low expression + not so essential*).

### 3.8. GO and KEGG pathway analysis 

**Figure 8:**
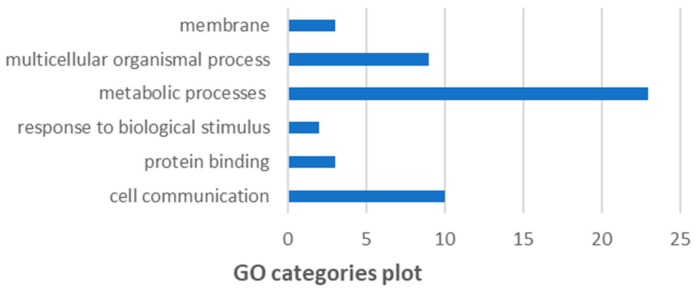
Gene set enrichment analysis of target genes based on GO categories.

As per GO analysis (Figure 8), top functions predicted are as follows: cell communication (10/50), protein binding (3/50), response to biological stimulus (2/50), metabolic processes (23/50), multicellular organismal process (9/50), membrane (3/50). Further, KEGG pathway enrichment analysis represents the active participation of the expressed gene sets into multiple KEGG pathways (Table 3). This analysis was based on Globaltest algorithm predicting relationship between biological response variable and metabolic pathway based on linear model computing *p*-statistic score. Further, *p*-values were adjusted using Benjamini and Hochberg’s False Discovery rate (FDR). 

## 4. Discussion

Carcinogenesis is the fallout of aberrant changes in signal transduction pathways fueled by both genetic and epigenetic alterations. Epigenetic alterations are increasingly being acknowledged as an early and ongoing alteration that aids in cancer initiation and progression. In this study, genistein was found to modulate the expression and activity of several epigenetic enzymes as well as various molecular targets. MTT assay established the EC_50_ of genistein in HeLa cells as 100 µM in 24 h [17]; therefore, 50 µM genistein treatment corresponding to 80% cell viability in 24 h and 55% viability in 48 h was used in this study. 

Genistein causes dysregulation of the cell cycle. *CCNB1, CCNB2, CCND3* are required for the cell to cross G2-M checkpoint; while Cyclin dependent kinase inhibitor 2D (CDKN2D) and Cyclin dependent kinase 2 (CDK2) are required for G1 arrest and interact with CCNB1 and CCNB2 [18]. Replicative immortality has been further achieved by the overexpression of telomerase reverse transcriptase (TERT), which determines telomere length and facilitates continued proliferation and evasion of apoptosis [19,20,21,22]. The cell cycle dysregulation mediated by genistein has been supported by the finding that *CCNB1, CCNB2, CCND3, CDKN2D* and *CDK2* are downregulated. Further, genistein downregulated *CDKN1A* gene expression, which aids in apoptosis. *TERT* expression was significantly downregulated by genistein hampering cellular immortality.

Genistein is known to restrict cell migration as evidenced through the scratch wound assay [17]. In order to understand the molecular changes behind these observations, various genes involved in this phenomenon were tested for changes in their expression. Migration is dependent on the balance between the pro-migration genes, *MMPs* and anti-migration genes, *TIMPs* [23]. *CDH1* is amongst the chief anti-migratory molecules and its role is supported by *SOCS1, COL1A1* while *TWIST1* is a prominent inhibitor of e-cadherin [24,25]. Genistein can impair the migration and invasion as it is found that it restores the expression of CDH1 and SOCS1. Further, pro-migratory proteins such as *TWIST1, MTA1* and *MMP14* are downregulated.

Several signaling pathways play a crucial role in attaining the features of cancer, influencing proliferation, migration and apoptosis. PI3K pathway is amongst the most central signaling pathways that influences carcinogenesis. *PI3KCA, PI3KCB, PI3KD* are important moieties in the signaling pathways with *AKT1* and *mTOR* being the chief effectors. These are therefore considered as important target molecules. Genistein influences this pathway by downregulating the expression of *PI3KCD* and *AKT*. Genistein has been reported to reduce the proliferation and induce the apoptosis in A549 lung cancer cells by inhibiting PI3K/AKT signaling pathways [26]. MAPK cascade induces the proliferation in tumour cells. These phytochemicals at the tested doses were found to modulate the expression of several genes in the MAPK cascade. Genistein reduced the expression of *MAPK3* and *MAP2K1*. Further, *PTPRR* expression was found to be upregulated. PTPRR is usually methylated in cervical cancer and as a MAP kinase pathway inhibitor, impacts various aspects [27]. 

Inflammation is accepted as a precursor and helper of carcinogenesis; with cytokines influencing various signaling pathways and encouraging the growth and spread of tumour [28]. Cytokines are also responsible for reduced efficacy of therapeutic agents [29]. Genistein downregulated *FOS* oncogene and *IL1A*, which will have an impact on inflammatory response. 

The expression of several genes in signaling pathways, oncogenes and tumour suppressor genes are altered in carcinogenesis and can be associated with alteration in epigenetic mechanisms. DNMT family of enzymes mediate methylation of DNA and are crucial players in epigenetic modification. Overexpression of *DNMT1, 3A* and *3B* has been reported in cervical cancer cells and is associated with disease progression [30]. *DNMT1, 3A and 3B* expression were downregulated marking a possible decrease in functional consequence (Figure 2). Genistein (50 μM) significantly decreased the enzymatic activity of DNMTs (Figure 3). Earlier, by using *in silico* docking studies we had shown that genistein could inhibit DNMT proteins [31]. DNMT1 is stabilized by PI3K-AKT pathway and is associated with DNA methylation [32]. In this study, it was found that genistein downregulated the expression of genes in the PI3K pathway and inhibited the biochemical activity of DNMT1. Downregulation of *DNMT*s and lowered activity has been shown to promote demethylation of the promoters of TSGs and aiding in reducing proliferation and inducing apoptosis [33].

HDAC1 and HDAC6 are overexpressed in cervical cancer and contributes in cancer progression, metastasis and angiogenesis [34,35]. Genistein showed the ability to reduce the activity of HDACs. Earlier, through *in silico* docking studies, we have shown that genistein is able to inhibit several HDAC enzymes [31]. Genistein downregulated the expression of *HDAC1, HDAC5* and *HDAC6* (Figure 2). Suppressing the activity and expression of HDACs resulted in increased expression of TSGs, reduced tumour growth and induced apoptosis [36].

Genistein modulated the expression of histone acetyltransferases (Figure 2). The function of ESCO2 is to block MMP2 and help apoptosis [37]. Genistein upregulates the expression of *ESCO2*, which is likely to impact its anti-migratory response. Genistein upregulates the expression of *CIITA*. CIITA upregulates the expression of class II major histocompatibility and contributes to immune response; it is usually methylated in cancer cells. 

H3K9 methyltransferases are enzymes that transfer methyl groups to lysine 9 of histone 3 causing repression of TSG expression. They are known to be over expressed and over-active in cervical cancer [38]. H3K9 methyltransferase activity was found to be significantly decreased after incubating with genistein. This repression contributes to the possible restoration of the expression of the silenced TSGs. DOT1/KMT4 protein contributes to cell proliferation, cell cycle progression, angiogenesis as well as DNA damage during G2 phase of the cell cycle [39,40,41]. *DOT1L* expression decreased with genistein treatment. Histone lysine methyltransferase, the SET domain containing protein 7 (*SETD7*), which functions as a TSG and causes p53 activation is usually suppressed by human papilloma virus (HPV) [42]. *SETD7* was found to be overexpressed after genistein treatment. Also, *SETD5* and *SETD6* were increased by genistein treatment. SETD6 helps in downregulation of Nuclear Factor kappa-light-chain-enhancer of activated B cells (NF-kB) mediated inflammation [43]. The function of *SETD5* has not yet been clearly determined. SUV39H1 affects the recruitment, localization and availability of DNMT3B and DNMT1, thereby affecting the methylation levels [44]. Genistein was able to downregulate this gene, which is a contributor to the cell’s methylation status. SUV420H1 methylates extracellular signal regulated kinase 1 (ERK1) that leads the activation of the oncogenic ERK1 pathway [45]; *SUV420H1* was downregulated by genistein.

The KDM family of genes are histone demethylases, which demethylate lysine 4 on histone 3; they contain Jumonji C domain. KDM1A (LSD1) is important for epithelial-to-mesenchymal transition (EMT) and promotes the progression of tumor [46]. Genistein reduces the expression of this pro-migration gene, which may contribute to their anti-migration effect. The structure and function of KDM5C is very similar to the highly tumourogenic KDM5A and KDM5B; it is overexpressed in cervical cancer and increased expression of this gene is associated with disease severity [47]. *KDM5C* expression was lowered by genistein. KDM6B overexpression in cervical cancer is mediated by HPV, and its suppression reduces proliferation [48]. *KDM6B* expression is lowered by genistein.

AURKA A and B are histone phosphorylases that are frequently overexpressed in cancer, and contribute to cell growth, G2-M checkpoint crossing, migration and regulation of the AKT pathway [47]. Genistein downregulated the expression of AURKA and AURKB. PAK1 silencing has been shown to decrease cell proliferation [49]. High PAK1 expression in cervical cancer is associated with its pathological features including angiogenesis, upregulation of MMP2, metastasis and poor prognosis [50,51]. It is therefore significant that genistein downregulates *PAK1* expression.

Ubiquitination tags ubiquitin onto proteins which are then degraded; ubiquitinases thereby influence several cellular processes. UBE2A is generally overexpressed in cervical cancer and is responsible for malignant transformation and chromosomal instability [52,53]. *UBE2A* expression was decreased by genistein.

The functional significance of the epigenetic modulation mediated by genistein can be gauged by methylation levels globally as well as specifically on promoters of certain TSGs. Genistein decreased the global DNA methylation level in a time-dependent manner (Figure 4). Genistein has been documented to lower global DNA methylation in breast cancer cells [54]. This is well correlated with the decrease in DNMT expression and activity. The therapeutic potential of genistein treatment was determined by its ability to reduce TSG promoter methylation viz. *APC, CDH1, GSTP1, RARB, RASSF1, MGMT, SOC51, CDH13, DAPK1, MLH1, PTEN, FHIT TIMP3* and *VHL*. These genes are frequently methylated in cervical cancer and associated with higher disease severity [55,56,57,58]. Several epigenetic modulatory agents have been known to lower TSG promoter methylation, restore transcription and contribute to apoptosis induction [16,59,60]. The antagonist of the Wnt pathway, *APC*, is often methylated and silenced and controls migration and apoptosis [61]. E-cadherin, (*CDH1*) is a fundamentally important gene that limits migration and is reported to be methylated in cervical cancer [62]. DAPK1 is frequently downregulated in cancer contributing to disease progression [57,63]. *FHIT* is a tumor suppressor gene, overexpressed in cancer; restoration of expression inhibits growth and induces apoptosis [64,65]. O-6-methylguanine-DNA methyltransferase (MGMT) is a DNA repair enzyme and has often been found to be methylated in several cancers, including cervical cancer [65,66]. MLH1 is involved in mismatch repair and cell cycle regulation through p21 and p73 [67]. MLH1 is methylated in cervical cancer and its methylation is associated with increased metastasis, recurrence and poor outcome [68,69]. PTEN inhibits the PI3K pathways and affects proliferation and survival [70]. *PTEN* is hypermethylated in cervical cancer and increases with the disease stage [71]. RARB is involved in cell proliferation and is usually methylated in several cancers including cervical cancer [72]. RASSF1A regulates various cellular processes including the cell cycle, mitotic arrest and apoptosis and is often methylated in cervical tumors [73,74,75]. *RUNX3* is a tumour suppressor gene that is consistently hypermethylated in cervical cancer [76]. Suppressor of cytokine signaling-1 (SOCS1), is a tumour suppressor gene and suppresses cytokine signaling and degrades HPV E7 protein. SOCS1 is hypermethylated in cervical cancer; restoration of its expression, increases Rb protein and suppresses cell proliferation [77,78]. TIMP3 is involved in restricting invasion and migration of cells and is usually methylated in cervical cancer [79]. VHL is involved in regulating the expression of several genes including *HIF1α* [54].

Further, Globaltest algorithm was utilized to identify the incurring relationship among statistically significant genes and biological pathways. A major participation is highlighted among cancer pathways. Network analysis in combination with topology analysis validates the essentiality of target genes in terms of degree centrality [80,81,82]. Co-expression and betweenness identifies treatment of genistein modulating the expression levels of several input genes. For example, active participation of histone deacetylases (*HDAC1, HDAC5* and *HDAC6*) in cervical cancer and significant degree made them vital for network maintenance. On genistein treatment, downregulation of these concerned epigenetic markers predicts their functionality loss and resultantly incomplete pathway. On the other hand, upregulation of *TP53* with best optimal network topological parameters made it essential for cell cycle maintenance and cancer prevention. Among 50 input target genes, a degree of 21, betweenness of 321.87 and expression of 1.78 depict sits essentiality for network maintenance. Further, its upregulation on genistein treatment represents its active role in angiogenesis inhibition and anticancer function. In accordance, GO and KEGG pathway analysis discerns the active participation of input gene sets in metabolic processes, multicellular organismal process and cell communication [83,84]. Functional association of the gene sets with the cellular machinery represents the essentiality of major portion of genes in underlying cancer pathways.

qRT-PCR results showed that genistein restored the expression of TSGs namely, *TP53, PTEN, CDH1, DAPK1, FHIT, RUNX3, SOCS1* and *RARb*. Other studies have reported similar findings by using an epigenetic modulatory agent [79,85,86,87]. However, there were no significant changes in *BRCA1, GSTP1, MLH1, RASSF1, TIMP3* and *VHL* gene expression at the transcript level, which could be caused by other transcription regulation. Genistein’s anti-carcinogenic effect, principally its effect on cell proliferation, migration and induction of apoptosis, may be explained by the modulation of various epigenetic and molecular targets and restoration of TSG expression. This study has shown that the effects of genistein is a multi-dimensional and has great potential to be developed as an effective anti-cancer target.

## Figures and Tables

**Table 1:** Genes modulated in HeLa cells treated by 50 μM genistein.

Molecular Targets	Transcriptional Expression Status	Genes Modulated by Genistein
Cell cycle regulation	Downregulated	*CCNB1, CCNB2, CCND3, CDKN2D, CDK2, CDKN1A*
Proliferation	Upregulated	*TP53, TP73, PTEN, TP53I3*
	Downregulated	*TERT*
Metastasis	Upregulated	*CDHI, SOCS1*
	Downregulated	*TWIST1, MMP14, MTA1*
PI3K Pathway	Downregulated	*PI3KCD, AKT1*
MAPK Pathway	Upregulated	*PTPRR*
	Downregulated	*MAPK3, MAP2K1*
Inflammation markers	Downregulated	*IL1A, FOS*
DNA methyltransferases	Downregulated	*DNMT1, DNMT3B DNMT3A*
Histone deacetylases	Downregulated	*HDAC5, HDAC1, HDAC6*
Histone acetylases	Upregulated	*CIITA, ESCO2*
	Downregulated	
Histone methylases	Upregulated	*SETD5, SETD7, SETD6*
	Downregulated	*DOT1L, SETD1B, SUV420H1, SUV39H1*
Demethylases	Downregulated	*KDM1A, KDM6B, KDM5C*
Histone phosphorylases	Downregulated	*AURKA, AURKB, PAK1*
Histone ubiquitinases	Downregulated	*UBE2A*
Tumour Suppressor Genes	Decreased promoter methylation	*APC, BRCA1, CDH1, CDH13, DAPK1, FHIT, GSTP1, MGMT, MLH1, PTEN, RARB, RASSF1, SOC51, TIMP3, VHL*
	Restored expression	*TP53, PTEN, CDH1, DAPK1, FHIT, RUNX3, SOCS1, TP53I3, TP73, RARβ*

**Table 2:** Topological parameters of target gene set.

S.No.	Gene Name	Degree	Betweenness	Expression
1.	*TP53*	21	321.87	1.78
2.	*HDAC1*	19	197.1	0.46
3.	*DNMT1*	9	70.1	0.31
4.	*CDKN1A*	8	50.55	0.5
5.	*SUV39H1*	7	40.5	0.5
6.	*CDK2*	7	39.98	0.45
7.	*KDM1A*	7	22.08	0.4
8.	*MAPK3*	5	74.08	0.4
9.	*AKT1*	5	44.48	0.45
10.	*CCNB1*	5	33.13	0.31
11.	*DNMT3B*	5	2.2	0.33
12.	*TP73*	5	1.42	5.27
13.	*HDAC5*	4	10.24	0.46
14.	*AURKA*	4	7.32	0.4
15.	*MTA1*	4	0	0.5
16.	*DNMT3A*	4	0	0.5
17.	*CIITA*	3	10.53	4.93
18.	*TWIST1*	3	2.95	0.5
19.	*HDAC6*	3	1.42	0.5
20.	*ESCO2*	3	1.07	13.93
21.	*MAP2K1*	2	7.39	0.47
22.	*PTEN*	2	7.06	2.1
23.	*TERT*	2	7.06	0.5
24.	*PAK1*	2	4.78	0.5
25.	*FOXO1*	2	4.47	1.89
26.	*CDH1*	2	2.23	2.76
27.	*AURKB*	2	0	0.44
28.	*CCNB2*	2	0	0.5
29.	*SETD7*	2	0	2.35
30.	*CCND3*	2	0	0.5
31.	*FOS*	2	0	0.37
32.	*DAPK1*	2	0	3.49
33.	*UBE2A*	2	0	0.38
34.	*TP53I3*	1	0	4.96
35.	*RUNX3*	1	0	3.76
36.	*SOCS1*	1	0	10.62
37.	*PTPRR*	1	0	4.03
38.	*KDM5C*	1	0	0.5

**Table 3:** KEGG pathway enrichment analysis of target genes under study. Total = total number of genes participating in pathway, Expected = minimum number of genes to be considered as statistically significant, Hits = total number of hits in pathway, False Discovery rate (FDR) = adjusted *p*-value.

KEGG Pathways	Total	Expected	Hits	*P*.Value	FDR
p53 signaling pathway	72	0.307	9	1.01e−11	3.21e−09
Cellular senescence	160	0.682	11	2.74e−11	4.36e−09
FoxO signaling pathway	132	0.563	10	9.84e−11	1.04e−08
HTLV-I infection	219	0.934	11	8.15e−10	6.48e−08
Endometrial cancer	58	0.247	7	3.36e−09	2.14e−07
Prostate cancer	97	0.414	8	4.89e−09	2.59e−07
Pathways in cancer	530	2.26	14	1.03e−08	4.68e−07
Bladder cancer	41	0.175	6	1.51e−08	5.59e−07
Melanoma	72	0.307	7	1.58e−08	5.59e−07
Progesterone-mediated oocyte maturation	99	0.422	7	1.48e−07	4.69e−06
Thyroid cancer	37	0.158	5	4.07e−07	1.18e−05
Glioma	75	0.32	6	6.1e−07	1.57e−05
Chronic myeloid leukemia	76	0.324	6	6.6e−07	1.57e−05
Cell cycle	124	0.529	7	6.93e−07	1.57e-05
Colorectal cancer	86	0.367	6	1.38e−06	2.85e−05
Viral carcinogenesis	201	0.857	8	1.43e−06	2.85e−05
Breast cancer	147	0.627	7	2.19e−06	4.09e−05
MicroRNAs in cancer	299	1.28	9	2.9e−06	4.98e−05
Endocrine resistance	98	0.418	6	2.97e−06	4.98e−05
Hepatitis B	163	0.695	7	4.36e−06	6.94e−05
Central carbon metabolism in cancer	65	0.277	5	7.09e−06	0.000107
Non-small cell lung cancer	66	0.282	5	7.64e−06	0.00011
Thyroid hormone signaling pathway	116	0.495	6	7.94e−06	0.00011
Renal cell carcinoma	69	0.294	5	9.53e−06	0.000126
Prolactin signaling pathway	70	0.299	5	1.02e−05	0.00013
Oocyte meiosis	125	0.533	6	1.22e−05	0.000149
Pancreatic cancer	75	0.32	5	1.44e−05	0.000169
Epstein-Barr virus infection	201	0.857	7	1.73e−05	0.00019
Proteoglycans in cancer	201	0.857	7	1.73e−05	0.00019
Measles	138	0.589	6	2.15e−05	0.000228
ErbB signaling pathway	85	0.363	5	2.65e−05	0.000272
Small cell lung cancer	93	0.397	5	4.1e−05	4e−04
Hepatitis C	155	0.661	6	4.15e−05	4e−04
T cell receptor signaling pathway	101	0.431	5	6.09e−05	0.00057
PI3K-Akt signaling pathway	354	1.51	8	9e−05	0.000818
